# Spinal Posture and Movement in Female Adolescents with Anorexia Nervosa

**DOI:** 10.3390/jcm15083054

**Published:** 2026-04-16

**Authors:** Munkh-Erdene Bayartai, Gabriella Tringali, Roberta De Micheli, Ilaria Grimoldi, Laura Abbruzzese, Anelise Sonza, Alessandro Sartorio

**Affiliations:** 1 Department of Physical and Occupational Therapy, School of Nursing, Mongolian National University of Medical Sciences, Ulaanbaatar 14210, Mongolia; 2 Experimental Laboratory for Auxo-Endocrinological Research, Istituto Auxologico Italiano, Istituto di Ricovero e Cura a Carattere Scientifico (IRCCS), 28824 Piancavallo-Verbania, Italy; g.tringali@auxologico.it (G.T.); r.demicheli@auxologico.it (R.D.M.); i.grimoldi@auxologico.it (I.G.); anelise.sonza@udesc.br (A.S.); sartorio@auxologico.it (A.S.); 3 Division of Auxology, Istituto Auxologico Italiano, Istituto di Ricovero e Cura a Carattere Scientifico (IRCCS), 28824 Piancavallo-Verbania, Italy; l.abbruzzese@auxologico.it; 4 Laboratorio de Desenvolvimento e Controle Postural (LADESCOP), Centro de Ciencias da Saude e do Esporte (UDESC/CEFID), Universidade do Estado de Santa Catarina, Florianopolis 88035-901, SC, Brazil

**Keywords:** anorexia nervosa, adolescent females, spinal posture, spinal movements

## Abstract

**Background**: Profound musculoskeletal alterations encompassing bones and soft tissues are common in people with anorexia nervosa (AN). This study aims to examine spinal posture and mobility in adolescents with AN, and to compare these outcomes with those obtained from normal-weight female controls. **Methods**: Spinal posture and movements were measured in 37 adolescents with AN and 31 normal-weight controls using the Idiag M360 scan tool, and between-group differences were analyzed using analysis of covariance. **Results**: Spinal postures and the lumbar-to-hip ratio were not different between subgroups. By contrast, AN had reduced thoracic (−21.8 degrees, *p* < 0.0001) and lumbar (−18.2 degrees, *p* < 0.0001) mobility in the frontal plane, as well as decreased hip flexion (−14 degrees, *p* = 0.001) and extension (−18.6 degrees, *p* < 0.0001) compared to the CG. **Conclusions**: Thoracic and lumbar spinal mobility, mainly in the frontal plane, and also hip mobility in the sagittal plane, are decreased in AN. These findings provide clinically relevant insights into spinal characteristics in adolescents with AN.

## 1. Introduction

Adolescent girls aged 15–19 represent the age group with the highest incidence of eating disorders [1]. Anorexia nervosa (AN) is a serious mental condition associated with persistent disturbances in eating behaviour, marked psychological pathology, and significant multisystem physical consequences [2], and it demonstrates the greatest mortality rate among psychiatric conditions [3]. Epidemiological evidence reports that despite advances in treatment, AN continues to have very high mortality rates, with individuals treated in inpatient settings experiencing an over five times higher mortality risk [4]. Prevalence estimates suggest that AN affects approximately 4% of females and 0.3% of males, with a notable and increasing incidence among individuals aged 15 years and younger [5]. Moreover, AN is a systemic disorder with widespread effects across multiple organ systems, among which the musculoskeletal system is frequently and severely impacted [6,7,8].

AN has profound adverse effects on the musculoskeletal system, encompassing muscles, bones, ligaments, tendons, and cartilage [6,7,8]. A characteristic feature in this population, particularly among females, is reduced bone mineral density and osteoporosis, which substantially elevate fracture risk [6,7]. While most research has focused on skeletal fragility, evidence regarding soft tissue involvement remains scarce. Nevertheless, a recent study reported a higher rate of injuries involving cartilage and ligaments among individuals with AN [9]. Furthermore, AN is associated with decreased muscle mass and diminished energy expenditure compared with unaffected individuals, although the implications of these changes for physiological decline remain inadequately understood [10]. Malnutrition in anorexia nervosa leads to muscle dysfunction, manifested as skeletal myopathy and reduced muscle performance [8]. These findings highlight the necessity of a comprehensive understanding of musculoskeletal alterations in AN to inform the development of targeted interventions aimed at optimizing musculoskeletal health and functional outcomes.

AN has been associated with structural and functional alterations of the spine [11]. Changes in spinal posture and movements are associated with musculoskeletal consequences. For example, altered spinal kinematics and reduced lumbar mobility are commonly related to low back pain [12]. Longitudinal research has indicated that reduced spinal movement in the frontal plane, along with a reduction in lumbar lordosis, heightens the likelihood of developing low back pain [13]. Furthermore, previous investigations of spinal posture and mobility in adults with AN have revealed that the condition is linked to changes in spinal alignment and movement patterns [11]. Nevertheless, the association between AN and spinal posture and movements in adolescents remains unclear. Therefore, the present study is focused on investigating spinal posture and mobility in adolescents with AN and comparing these outcomes with those obtained from age-matched, normal-weight female controls.

## 2. Materials and Methods

### 2.1. Participants

The current cross-sectional study involved 68 female adolescents aged 10–18 years. Participants with AN (no. 37, mean age + SD: 15.8 + 1.5 years; BMI: 15.6 + 1.9 kg/m^2^) were hospitalized at the Division of Auxology, Istituto Auxologico Italiano, IRCCS, Piancavallo (VB), Italy, for a 4-week period of nutritional rehabilitation. The diagnosis of anorexia nervosa was established in accordance with the DSM-5 criteria, using the Structured Clinical Interview–Clinical Version (SCID-5 CV). Normal-weight, age-matched healthy female controls without a history of spinal disorders (CG, mean age + SD: 15.9 + 1.8 years; BMI + SD: 20.9 + 1.0 kg/m^2^) were recruited from the research, administrative, and medical staff.

All participants provided written informed consent. Individuals with comorbid psychiatric disorders as defined by DSM-5, or with medical conditions that could interfere with participation, were excluded. Participants with AN were recruited at their initial visit to our third-level centre, with prior engagement limited to outpatient care.

The study protocol received approval from Ethics Committee No. 5 of the Lombardy Region in Milan, Italy (registration number 137/25; approval date April 4, 2025; research order code 01C516). All procedures complied with the principles of the 1975 Declaration of Helsinki and its later amendments, or with comparable ethical guidelines.

Prior to study enrollment, all potential participants were fully informed about the aim of the study and methodological procedures. Adolescents who agreed to participate voluntarily, together with their legal guardians, provided written informed consent in accordance with ethical requirements. After enrollment, demographic and clinical data relevant to the study population were collected via self-reported questionnaires.

### 2.2. Measurements

Body weight was assessed using a calibrated scale accurate to 100 g. Height was measured in the standing position using a Harpenden stadiometer (Holtain Limited, Crymych, Dyfed, UK). BMI was calculated as weight in kilograms divided by height in metres squared.

Spinal posture, spinal mobility, and hip movements were determined using the Idiag M360 scanning system (Idiag, Fehraltorf, Switzerland) [14]. Spinal posture was evaluated with participants standing upright, while spinal mobility was measured during spinal flexion, extension, and lateral bending. The Idiag M360 provides a safe, accurate, and radiation-free assessment of spinal alignment and movement. Vertebral joint and sacral angles, defined as the sacrum’s orientation relative to the vertical axis, are measured through computer-assisted analysis. During assessment, two integrated rolling wheels trace the spinous processes from C7 to S3. The device records segmental spinal alignment and motion in both the sagittal and frontal planes at a sampling rate of 150 Hz [14,15]. The acquired signals are digitized via an analogue-to-digital converter and transferred to a computer for further analysis.

The mobility of the spine and hips were evaluated in the sagittal and frontal planes. Tasks included forward bending, extension, and side bending from a standing position. After a research team member demonstrated the tests, participants performed each task once. Spinal flexion was assessed with participants maintaining an upright standing position before bending forward to the maximum range achievable within comfort, while keeping the knees fully extended. Spinal extension was subsequently evaluated by asking participants to extend the trunk posteriorly to the maximum comfortable range, ensuring that the knees remained fully extended during the task. Participants performed side bending, sliding one hand along the same-side leg to their comfortable limit, while carefully avoiding any accompanying trunk flexion or extension [11,14,16,17]. The device has demonstrated good validity and reliability for evaluating spinal posture and mobility in different study populations [14,15,18,19]. Validation studies comparing spinal curvature measurements obtained from radiographic examinations with those derived from the Idiag M360 have reported strong correlations [14,18]. In addition, spinal mobility documented in the earlier study show strong agreement with those obtained using the Idiag M360 [15]. A comparative study examining lumbar flexion demonstrated that segmental and overall lumbar mobility, as measured radiographically, closely matched the estimates provided by the device [19]. Moreover, the Idiag M360 demonstrated moderate-to-good reliability in evaluating spinal alignment and motion patterns among non-obese people [15].

### 2.3. Data Processing

For each spinal parameter, range of motion in the sagittal and frontal planes was calculated as the difference between values recorded in the standing position and the corresponding end-range measurements for each segment. Kyphotic and lordotic angles were defined with respect to the horizontal plane, whereas thoracic and lumbar flexion and extension for each spinal segment were measured in the sagittal plane (frontal axis). Lateral flexion of the thoracic and lumbar spinal segments was defined in the frontal plane (sagittal axis). Spinal posture and ranges of motion, including lumbar and thoracic regions, were obtained by summing the five segmental values for the lumbar spine and the twelve segmental values for the thoracic spine. The lumbar-to-hip ratio was calculated by dividing the lumbar range of motion by the total lumbar plus hip ranges during trunk flexion in the sagittal plane.

### 2.4. Statistical Analysis

Statistical analyses were conducted using R software (version 4.5.0) [20]. Descriptive statistics included means, standard deviations, sample sizes, and percentages for variables such as age, sex, spinal posture, spinal mobility, hip motion, and the lumbar-to-hip ratio. The Shapiro–Wilk test was used to check normality of the data. Subgroup comparisons were conducted using independent-samples *t*-tests for normally distributed variables and Wilcoxon rank-sum tests for non-normally distributed variables to evaluate differences between the AN group and CG. Analysis of covariance (ANCOVA), adjusted for age, was employed to investigate group differences in spinal parameters. Post hoc pairwise comparisons were performed using the “emmeans” package following ANCOVA analyses [21]. Statistical significance was defined as a *p*-value of 0.05 or less.

## 3. Results

**Figure 1:**
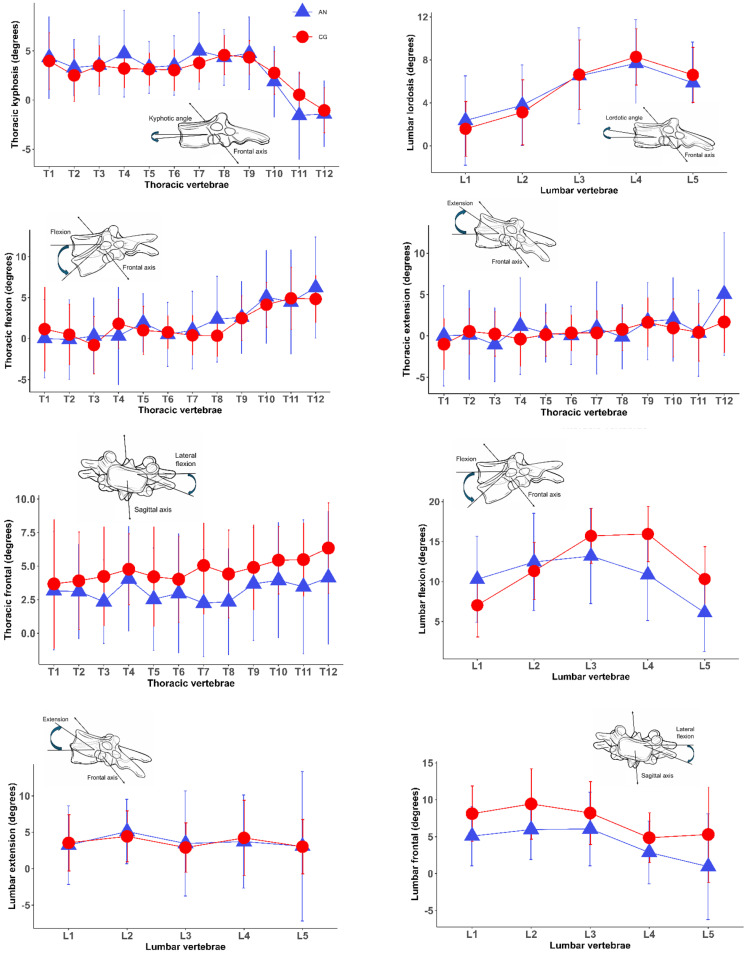
Segmental posture and mobility of the spine in the subgroup of individuals with AN and in normal-weight controls (mean and standard deviations). Positive values correspond to kyphosis/flexion, while negative values correspond to lordosis/extension. CG: normal-weight controls, AN: individuals with anorexia nervosa.

The current study involved 68 female adolescents. The demographic and anthropometric characteristics of the study population are reported in Table 1. Mean age and height were similar in the AN and CG subgroups, while individuals with AN had lower body weight and BMI, as expected. Reduced segmental spinal mobility was more frequently observed in the frontal plane, and hip mobility in the sagittal plane, in individuals with AN than in the CG (Figure 1, Table 2).

### Comparison of Spinal Posture and Mobility Between Individuals with AN and CG

Individuals with AN had reduced thoracic and lumbar mobility in the frontal plane (lateral flexion) compared to the CG. Female adolescents with AN also showed less lumbar flexion (−6.3 degrees) than the CG. The hip movements in the sagittal plane (flexion and extension) were decreased compared to those of normal-weight controls. The largest difference in spinal mobility observed between the AN group and CG was the thoracic lateral flexion, with a decrease of 21.8 degrees. No differences were observed in the lumbar-to-hip ratio between the two groups (Table 2).

## 4. Discussion

The aim of the current study was to explore spinal posture and mobility in female adolescents with AN, compared to an age- and sex-matched normal-weight control group (CG). The main finding was that AN in a pediatric population was associated with alterations in spinal mobility. Female adolescents with AN showed reduced spinal and hip mobility compared to female individuals of normal weight. By contrast, no differences in spinal posture were observed between individuals with AN and CG. These findings provide clinically relevant insights into spinal characteristics in adolescents with AN and underscore the importance of considering these factors in the musculoskeletal assessment of adolescents with this clinical condition.

AN was associated with reduced spinal mobility in this pediatric study population, characterized by reduced lateral flexion of the thoracic and lumbar spine and decreased lumbar flexion. Studies examining spinal mobility and posture in subjects with AN are limited. A recent study by our group in adults with AN has identified decreased thoracic and lumbar lateral flexion and lumbar flexion, although the results did not reach statistical significance [11]. These findings suggest that reduced spinal mobility observed in adolescents with AN may also be present in adults with the condition. Nevertheless, this hypothesis requires further investigation in future studies, given the scarcity of studies addressing this topic.

Furthermore, the reduced spinal mobility observed in adolescents with AN in the present study may represent a biomechanical disadvantage, potentially increasing susceptibility to stress-related spinal disorders. Previous biomechanical research has shown that greater thoracic flexibility reduces mechanical loading on the lumbar spine, potentially helping protect against stress-related spinal conditions [22]. Individuals with AN appear to be susceptible to back pain, as muscle pain and cramps are commonly reported and are often associated with deficiencies in potassium, phosphorus, and magnesium [23]. Moreover, prospective research has shown that reduced frontal-plane spinal mobility predicts the onset of low back pain [13]. In the present study, adolescents with AN showed reduced thoracic and lumbar frontal-plane movements compared with normal-weight controls, suggesting that these impairments could be one of several factors potentially associated with low back pain in this population. However, reduced spinal mobility in individuals with AN could also be the result of structural weakness due to malnutrition, such as weakened intervertebral discs and diminished bone strength. Given the paucity of the existing literature, further research is needed to investigate the key features of AN in relation to muscle loss and spinal mobility, to confirm the present findings.

Individuals with AN demonstrated reduced hip extension compared with normal-weight controls, while no significant differences were found in spinal posture. Research has consistently indicated that individuals with AN show decreased hip strength, potentially due to metabolic imbalances and reduced bone mineral density [24,25]. Such impairments may explain the observed restriction in hip extension in the present study. Contrary to the alterations in spinal posture we found in adult females with AN [11], no differences in spinal posture were observed between adolescents with AN and normal weight controls. Individuals with AN showed reduced lumbar lordotic curvatures, although the difference was not statistically significant, suggesting that this alteration may still contribute to musculoskeletal strain and pain, such as low back pain, by increasing stress on the vertebrae and intervertebral discs. Although these findings suggest that spinal posture may change with ageing in individuals with AN, further investigation is required to confirm this hypothesis.

We acknowledge several limitations in the present study. First, the cross-sectional nature of the study prevents conclusions about causality between AN and spinal motion alterations. To date, the discrepancies between adolescent and adult findings remain uncertain. Participants performed the ROM and functional tests only once, potentially reducing reliability and increasing measurement error. Furthermore, any explanation related to growth, ageing, or postural changes remains hypothetical and cannot be confirmed by the current study. Second, the measurement device was limited to assessing spinal posture and movement in the sagittal and frontal planes, preventing evaluation of spinal rotation in the horizontal plane. Spinal flexibility in isolation does not appear to be a reliable indicator of back health, as it is influenced by complex interactions among factors such as soft-tissue integrity, core muscle strength, and the cumulative mechanical demands of repetitive movements performed with or without load. Finally, given that the study involved a relatively small sample and a post hoc power analysis indicated it was underpowered to detect the observed differences in spinal mobility (power = 65%), the findings should be validated in larger cohorts that include participants of both sexes and a wider age range.

## 5. Conclusions

In conclusion, adolescent females with AN demonstrated reduced thoracic and lumbar mobility compared with age- and sex-matched normal-weight controls. These findings provide clinically relevant insights into spinal characteristics in adolescents with AN and underscore the need to consider these factors in the musculoskeletal assessment of these subjects.

## Figures and Tables

**Table 1:** Main characteristics of the study population.

Variables	AN (*n* = 37)	CG (*n* = 31)	*p*-Value
Age (years)	15.8 (1.5)	15.9 (1.8)	0.83 ^W^
Weight (kg)	40.3 (6.9)	55.5 (3.2)	<0.0001 ^W^
Height (cm)	160.5 (8.0)	162.6 (4.0)	0.82 ^W^
Body mass index (kg/m^2^)	15.6 (1.9)	20.9 (1.0)	0.002 ^W^

Data are presented as mean ± standard deviations; *p*-value: the differences between the two groups were evaluated using Wilcoxon’s rank-sum test. ^W^ Wilcoxon’s rank-sum test.

**Table 2:** Spinal posture and mobility of the study population.

Variables	AN *n* = 37		CG *n* = 31		Differences in Spinal Posture and Mobility (95% CI)	*p*-Value
	EMM	SE	EMM	SE		
Spinal postures						
Thoracic kyphotic curvatures (Th1–12)	35.8	1.5	36.3	1.6	−0.5 (−4.9 to 3.8)	0.81
Lumbar lordotic curvatures	26.2	1.6	28.4	1.8	−2.2 (−6.9 to 2.7)	0.37
Sacral angle	15.4	1.5	16.3	1.7	−0.8 (−5.5 to 3.7)	0.70
Spinal mobility						
Thoracic (°)						
Flexion	24.7	1.9	21.4	2.2	3.3 (−2.6 to 9.2)	0.26
Extension	10.5	2.8	4.3	3.1	6.3 (−2.1 to 14.6)	0.14
Lateral flexion	38.0	3.0	59.8	3.3	−21.8 (−30.8 to −12.8)	<0.0001 *
Lumbar (°)						
Flexion	52.9	2.0	59.2	2.2	−6.3 (−12.2 to −0.3)	0.03 *
Extension	18.6	2.2	17.8	2.4	0.8 (−5.6 to 7.3)	0.79
Lateral flexion	21.0	2.1	39.2	2.3	−18.2 (−24.5 to −11.8)	<0.0001 *
Hip mobility						
Hip (°)						
Flexion	38.3	2.8	52.3	3.1	−14.0 (−22.4 to −5.6)	0.001
Extension	2.7	2.7	21.3	3.0	−18.6 (−28.6 to −10.4)	<0.0001 *
Lumbar-to-hip ratio	0.6	0.02	0.5	0.02	0.04	0.18

AN, individuals with anorexia nervosa; CG, normal-weight control group; *p*-value (adjusted for age): the differences between the two groups. The estimated marginal means (EMMs) of the total spinal segment range of motion across spinal regions are shown. SE = standard error; CI = confidence interval. * *p* < 0.05.

## Data Availability

Raw data will be available on www.zenodo.org after the acceptance of the manuscript upon reasonable request to the corresponding author.
